# Editorial: Patient-centered communication skills for health professions education and healthcare

**DOI:** 10.3389/fpubh.2023.1311905

**Published:** 2023-11-07

**Authors:** Astrid Pratidina Susilo, Jill Benson, Rosaria Indah, Mora Claramita

**Affiliations:** ^1^Department of Medical Education and Bioethics, Faculty of Medicine, Universitas Surabaya, Surabaya, Indonesia; ^2^Discipline of General Practice, University of Adelaide, Adelaide, SA, Australia; ^3^Department of Medical Education, Faculty of Medicine, Universitas Syiah Kuala, Banda Aceh, Indonesia; ^4^Department of Medical Education and Bioethics, Faculty of Medicine, Pubic Health, and Nursing, Universitas Gadjah Mada, Yogyakarta, Indonesia

**Keywords:** communication skills, patient-centered, health professional, respect, learning

Von Fragstein et al. (1) published an article on the consensus statement of communication skills training for undergraduate medical curricula. This article, never too old to revisit, presented a comprehensive communication curriculum using a visualization of the “wheel.” This Communication Curriculum Wheel showed that throughout the journey of becoming health professionals and beyond, students need to master different tasks of communication skills (e.g., building rapport, exploration, explanation and planning), apply them in various situations of patient encounters (e.g., age-specific communication, sensitive issues, dealing with uncertainty, handling mistakes), able to use different media in communication (e.g., face-to-face, telephone, written), and involve different stakeholders (e.g., family or other health professionals). At the very center of the wheel, the authors placed “respect for others,” which strongly urges all health professionals to reflect that respect is the basis of all communication (1). In health care, we translate respect as patient-centered communication.

Our Research Topic elaborates on patient-centered communication skills, which we believe are the key to better health outcomes. Along with professionalism and lifelong learning, communication skills are essential for health professionals, including soft and complex abilities. Moreover, communication skills should be sensitive to the cultural background and the context of society, nurtured during health professions education, and continuously strengthened throughout our professional lives (2, 3). This Research Topic brings together seven outstanding contributions from different cultural contexts around the globe, both from Western and Eastern cultures. From the original target of four publication in this series, about 50% of the submitted manuscript were accepted, 14 submitted 7–7 accepted-rejected. The included studies explored various aspects of patient-centered communication in clinical practice and education as described below:

Li et al. developed a scoping review on Shared Decision Making (SDM) in mainland China, a country with a history of doctor-led solid decision-making models, where patients traditionally rely on doctors' discretion. They reported challenges in implementing SDM and called out for policy support. Furthermore, this review underlined that it is essential to tailor the SDM to the culture, which is strongly filial, to facilitate patients' involvement. Consequently, a specific SDM framework tailored to the culture is essential.

Wang et al. conducted an extensive survey of 3,000 participants from 103 hospitals in different regions in China which studied the relationship between patients' attribution to negative medical situations and health professionals' communication and humanization. Humanization in health care emphasizes respect for patient's dignity and autonomy. Patients with biopsychosocial and spiritual dimensions should be treated as a whole human being, not only viewed as diseases or symptoms. This cross-sectional study reported that patients' perception of medical staff's humanization in health care and their communication skills predicted how patients perceive their relations with their physicians when potential conflicts arise. Therefore, the authors called for enhancing communication skills training for health professionals to show respect through verbal and non-verbal communication.

In this Research Topic, we advocate that patient-centered communication can go beyond patient encounters. This perspective is elaborated in the systematic review by van der Voorden et al. on patient engagement in value-based healthcare. This review underlined the importance of inviting patients, as the most critical stakeholders in healthcare, to climb to a higher level of partnership. Patients are our partners in co-designing innovation to enhance patient-centered care in health and become involved in driving improvement based on their value. Nevertheless, this review reported that patient engagement in value-based healthcare is still in an early phase, and thus, there is vast opportunity for innovation in this area.

This Research Topic presented several studies on the education of patient-centered communications. A study by Yu et al. from the Netherlands investigated roleplays for non-Dutch students in medical consultation using Dutch as their second language. The authors reported that playing roles as both patients and doctors could increase a learners' motivation and feelings of relatedness and finally influence their competence. The results add to the evidence of the benefit of roleplays in learning, and how they can be transferred to different contexts using patient encounter simulation.

Another study from Australia by Dewi et al. underlined the importance of direct observation and feedback to improve medical students' communication skills in clinical settings. In the clinical environment, communication skills training is embedded in daily patient encounters and not trained separately. Therefore, the roles of clinical teachers to facilitate the learning process are vital.

In two articles, we gave special attention to communication in telemedicine. Telemedicine is a part of future medical practice, so preparing future doctors to communicate in this context is essential. Findyartini et al. reported how self-reflective writing can stimulate students to internalize the importance of patient-centered communication. During a four-week telemedicine-based course, students were assigned to monitor COVID-19 patients undergoing self-isolation. This qualitative study found that this learning experience enables students to reflect on the pearls and pitfalls in communication with patients in the telemedicine context and helps students develop the necessary skills.

Festl-Wietek et al. developed a training to prepare students for written asynchronous online conversations. In line with previous theories in communication, the authors argued that online communication should be appreciative, emphatic, and authentic. Nevertheless, there is an additional challenge in telemedicine. The relevance of non-verbal aspects in human interaction might be lost as the sender and receivers of communication do not see each other. The training has led students to provide more appreciative responses toward patients in written online communication.

In conclusion, the diverse range of issues in this Research Topic showcases the complexity of this field and the need for innovative solutions to address emerging challenges in different cultural, clinical, and educational contexts. Although scientific journals continuously discuss patient-centered communication skills, evidence shows that gaps exist in different areas, and therefore, advocacy toward patient-centered communication skills and calls for fresh innovations are still urgent. As practitioners, teachers, researchers, or policymakers, we can continue collaborating to advance this ever-evolving field further and enhance the respect for patients' dignity and autonomy.

## Author contributions

AS: Writing – original draft. JB: Writing – review & editing. RI: Writing – review & editing. MC: Writing – review & editing.
